# Data analysis protocol for early autonomic dysfunction characterization after severe traumatic brain injury

**DOI:** 10.3389/fneur.2024.1484986

**Published:** 2024-12-24

**Authors:** Kejun Dong, Vijay Krishnamoorthy, Monica S. Vavilala, Joseph Miller, Zeljka Minic, Tetsu Ohnuma, Daniel Laskowitz, Benjamin A. Goldstein, Luis Ulloa, Huaxin Sheng, Frederick K. Korley, William Meurer, Xiao Hu

**Affiliations:** ^1^Center for Data Science, Nell Hodgson Woodruff School of Nursing, Emory University, Atlanta, GA, United States; ^2^Critical Care and Perioperative Population Health Research (CAPER) Unit, Department of Anesthesiology, Duke University, Durham, NC, United States; ^3^Department of Anesthesiology, School of Medicine, Duke University, Durham, NC, United States; ^4^Department of Anesthesiology and Pain Medicine, University of Washington, Seattle, WA, United States; ^5^Department of Emergency Medicine, Henry Ford Hospital, Detroit, MI, United States; ^6^Department of Emergency Medicine, School of Medicine, Wayne State University, Detroit, MI, United States; ^7^Faculty of Biotechnology and Drug Development, University of Rijeka, Rijeka, Croatia; ^8^Department of Neurology, Duke University Medical Center, Durham, NC, United States; ^9^Department of Biostatistics and Bioinformatics, School of Medicine, Duke University, Durham, NC, United States; ^10^Department of Emergency Medicine, University of Michigan, Ann Arbor, MI, United States; ^11^Department of Neurology, University of Michigan, Ann Arbor, MI, United States

**Keywords:** severe traumatic brain injury (sTBI), early autonomic dysfunction (eAD), electrocardiogram (ECG), arterial blood pressure (ABP), physiological waveform

## Abstract

**Background:**

Traumatic brain injury (TBI) disrupts normal brain tissue and functions, leading to high mortality and disability. Severe TBI (sTBI) causes prolonged cognitive, functional, and multi-organ dysfunction. Dysfunction of the autonomic nervous system (ANS) after sTBI can induce abnormalities in multiple organ systems, contributing to cardiovascular dysregulation and increased mortality. Currently, detailed characterization of early autonomic dysfunction in the acute phase after sTBI is lacking. This study aims to use physiological waveform data collected from patients with sTBI to characterize early autonomic dysfunction and its association with clinical outcomes to prevent multi-organ dysfunction and improving patient outcomes.

**Objective:**

This data analysis protocol describes our pre-planned protocol using cardiac waveforms to evaluate early autonomic dysfunction and to inform multi-dimensional characterization of the autonomic nervous system (ANS) after sTBI.

**Methods:**

We will collect continuous cardiac waveform data from patients managed in an intensive care unit within a clinical trial. We will first assess the signal quality of the electrocardiogram (ECG) using a combination of the structural image similarity metric and signal quality index. Then, we will detect premature ventricular contractions (PVC) on good-quality ECG beats using a deep-learning model. For arterial blood pressure (ABP) data, we will employ a singular value decomposition (SVD)-based approach to assess the signal quality. Finally, we will compute multiple indices of ANS functions through heart rate turbulence (HRT) analysis, time/frequency-domain analysis of heart rate variability (HRV) and pulse rate variability, and quantification of baroreflex sensitivity (BRS) from high-quality continuous ECG and ABP signals. The early autonomic dysfunction will be characterized by comparing the values of calculated indices with their normal ranges.

**Conclusion:**

This study will provide a detailed characterization of acute changes in ANS function after sTBI through quantified indices from cardiac waveform data, thereby enhancing our understanding of the development and course of eAD post-sTBI.

## 1 Introduction

Traumatic brain injury (TBI) is a heterogeneous condition that leads to significant injury-related disability and mortality (1). TBI not only impacts the brain but also results in extracranial multi-organ dysfunction, leading to secondary brain injuries and poor clinical outcomes (2). In 2014, TBI accounted for 2.8 million Emergency Department visits, 288,000 hospitalizations, and 56,800 deaths (3). One-third of survivors of hospitalization suffer long-term disability (3). The severity of TBI falls into three severity classifications based on the Glasgow Coma Scale (GCS) score following resuscitation: mild (GCS 13–15), moderate (GCS 9–12), and severe (GCS < 9), contingent upon criteria such as the duration of loss of consciousness, post-traumatic amnesia (4). However, variation in presentation, hospital course, and outcomes within each severity category is significant. Individuals initially diagnosed with mild or moderate TBI upon hospital admission may deteriorate, necessitating escalation in TBI management (5–7).

### 1.1 Extracranial organ dysfunction and sympathetic activation following TBI

The pathophysiology of extracranial organ dysfunction following TBI is characterized by complex neuroendocrine and inflammatory cascades. The initial brain injury triggers an intense sympathetic activation, leading to a massive release of catecholamines and inflammatory mediators (8). This autonomic response creates a paradoxical situation: while the acute elevation in sympathetic tone may initially serve as a protective mechanism to maintain cerebral perfusion despite increased intracranial pressure, its persistence can lead to detrimental systemic effects (9).

The sustained sympathetic hyperactivity, which can persist for up to 10 days post-injury, results in circulating catecholamine levels that may be up to 10-fold higher than normal (8). This catecholamine surge serves to maintain cerebral perfusion pressure in the face of rising intracranial pressure, but simultaneously can induce direct cardiac injury through catecholamine-mediated myocardial damage, systemic inflammatory responses, immune system dysfunction and metabolic derangements. These mechanisms help explain why 22.3% of patients with isolated moderate-to-severe TBI demonstrate left ventricular dysfunction as early as 24 h after injury (10). When this cardiac dysfunction occurs in the context of impaired cerebral autoregulation (present in 40% of moderate-to-severe TBI cases), it can create a vicious cycle: decreased cardiac output leads to systemic hypotension, which further compromises cerebral blood flow and brain perfusion.

Recent research using clinical scoring systems, particularly the Sequential Organ Failure Assessment (SOFA), has quantified the burden of multi-organ dysfunction following TBI. Studies have shown that nearly 40% of patients with moderate to severe TBI develop multi-organ dysfunction syndrome (MODS) within the first 10 days of hospitalization, with predominant involvement of the cardiopulmonary systems (11). Furthermore, examination of SOFA scores within the initial 72 h post-admission revealed that 252 patients (68%) with moderate to severe TBI developed early MODS (12). Understanding these pathophysiological mechanisms and their temporal evolution is crucial for predicting MODS risk, providing early warning of clinical deterioration, and guiding therapeutic interventions.

The relationship between intracranial pressure (ICP), autonomic function, and hemodynamics in TBI follows distinct temporal patterns. In the acute phase, elevated ICP triggers an intense sympathetic response with increased catecholamine release, leading to systemic vasoconstriction and hypertension (13). This response can become particularly problematic in cases of refractory intracranial hypertension. As demonstrated in (14), refractory ICP elevations are associated with a marked dysregulation of autonomic function, characterized by persistent sympathetic hyperactivation, reduced heart rate variability, impaired baroreflex sensitivity and development of refractory arterial hypertension. These autonomic alterations can persist even after ICP normalization, suggesting a more complex pathophysiology than previously recognized. In the subacute phase, this initial sympathetic surge may be followed by autonomic exhaustion, leading to vasodilation and hypotension. Understanding these temporal patterns and their relationship to autonomic dysfunction is crucial for appropriate hemodynamic management in TBI patients.

### 1.2 Heart rate variation with autonomic dysfunction

ANS critically regulates heart and vascular system functions by utilizing sympathetic and parasympathetic fibers directed toward the heart, alongside sympathetic fibers targeting the vessels (15). One hand, the release of norepinephrine from autonomic sympathetic fibers in heart induces positive inotropic and chronotropic effects through the activation of *β*-adrenoceptors. The other hand, acetylcholine released by parasympathetic fibers leads to negative inotropic and chronotropic effects by stimulating muscarinic receptors (16). In addition, the autonomic nervous system influences arterial blood pressure (ABP) and heart rate by producing oscillations in these cardiovascular parameters that occur at specific frequencies (16). In frequency domain analysis, the high frequency (HF, 0.15–0.4 Hz) component primarily reflects respiratory sinus arrhythmia (RSA) and is predominantly influenced by parasympathetic modulation, though respiratory parameters such as rate and tidal volume can significantly affect its interpretation. The low frequency (LF, 0.04–0.15 Hz) component and LF/HF ratio, previously considered markers of sympathetic activity or sympathovagal balance, actually represent an intricate interplay of both sympathetic and parasympathetic influences on cardiac autonomic control. Several factors contribute to LF power, including baroreflex-mediated autonomic fluctuations, central oscillations in sympathetic nerve activity, and mechanical effects of breathing at lower frequencies.

However, in the severe traumatic brain injury (sTBI) population, the interpretation of frequency-domain HRV metrics is complicated by several factors. Mechanical ventilation, often required for sTBI patients, can artificially alter respiratory patterns, impacting HF power and respiratory-related heart rate modulation. Additionally, the use of sedation and analgesic medications, common in sTBI management, can directly affect autonomic regulation and HRV patterns. Intracranial pressure fluctuations, characteristic of sTBI, can independently influence autonomic regulation and cardiovascular variability. Furthermore, hemodynamic management strategies involving vasopressors and fluid management can affect blood pressure variability and consequent autonomic responses. Baguley et al. investigated dysautonomia following sTBI based on the HRV analysis, finding significant differences in HRV among TBI patients, controls, and between dysautonomia and non-dysautonomia subjects (17). Froese et al. assessed the physiological relationship between pressure reactivity index (PRx) and HRV, BP, and baroreflex sensitivity (BRS) using time-series statistical methodologies in sTBI patients. They demonstrated a stronger connection between BRS, HRV, and PRx, indicating sympathetic autonomic response related to cerebrovascular reactivity derangements (18). Therefore, while these frequency-domain metrics provide valuable insights into autonomic regulation, their interpretation in sTBI patients should account for these confounding factors and be integrated with other autonomic assessment measures for a more comprehensive evaluation of autonomic dysfunction.

### 1.3 Need for clinical tools to assess autonomic dysfunction in clinical care

Despite the high burden of extracranial clinical deterioration, multi-organ dysfunction, and early autonomic dysfunction following sTBI, there are no real-time clinical decision support (CDS) tools, early warning scores, guidelines, or studies to help clinicians predict which patients will develop MODS. The sTBI-related literature lacks robust and well-powered analysis of autonomic function using cardiac and ABP waveform data, often including only exploratory datasets. sTBI is associated with eAD, yet characterization of eAD and its impact on clinical outcomes, in large sTBI populations is lacking. To bridge this gap, this protocol outlines the waveform data analysis for eAD characterization post-sTBI using a large prospective cohort (collected within the confines of a multicenter randomized controlled trial). We propose examining granular cardiac waveform data to identify subtle changes in the ANS in patients following sTBI that precede visible clinical symptoms of MODS. These changes can serve as early indicators of autonomic dysfunction, which is a precursor to MODS. The measurable metrics from cardiac waveform analysis, such as HRV, BRS, and heart rate turbulence (HRT), can be integrated into predictive models. These models can then be used to develop CDS tools that help clinicians assess the risk of MODS development in real-time. Furthermore, the metrics from waveform analysis can be employed to create early warning scores that gage the risk of MODS, using established thresholds for parameters like HRV and BRS linked to adverse outcomes in sTBI patients. Therefore, this protocol for characterization of eAD using ECG and ABP waveforms offers a pathway toward the development of real-time CDS tools, predictive models, and clinical guidelines.

## 2 Methods

### 2.1 Recruitment

We will collect data from an ongoing multi-center randomized controlled trial examining the efficacy of a sTBI treatment strategy guided by both ICP and brain tissue oxygen (PbtO2) as compared to a strategy guided by ICP monitoring alone [Brain Oxygen Optimization in sTBI (BOOST), Phase 3, NCT03754114] (17). As part of the trial, the ECG waveform with four-leads and arterial blood pressure waveform data will be collected over the first 5 days of their intensive care unit (ICU) stay. Our ancillary study (AUTO-BOOST) will leverage this rich and granular waveform data to fully characterize autonomic dysfunction following sTBI.

We are sourcing data from this active clinical trial (BOOST), the trial has enrolled 550 patients. Since unexpected issues may arise during data collection, such as failures to collect data due to leads detaching or errors caused by device malfunctions, we estimate that approximately 50% of patients will have adequate cardiac telemetry data of sufficient quality for analysis. The Moberg monitor (CNS Monitor, Moberg ICU Solutions, Amber, Pennsylvania, United States) will capture and time-synchronize these analog data with other vital signs, ventilator data, and ICP/PbtO2 data, storing all data locally. A research team member will extract and upload the waveform data to a secure IBM cloud-based server. We will extract ABP, ECG data, and ventilator parameters from the Moberg device, deriving heart rate from the raw ECG tracing. We will use cleaned segments of stable recordings within 24 h of ICU admission for analysis.

### 2.2 Data analysis

Based on data from the currently recruited 550 patients, we found some incomplete ECG recordings of short duration, necessitating data analysis based solely on the ABP signal. To address this issue, we will conduct the eAD characterization on two fronts: one from both ECG and ABP waveforms, and the other from only ABP waveforms.

**Figure 1:**
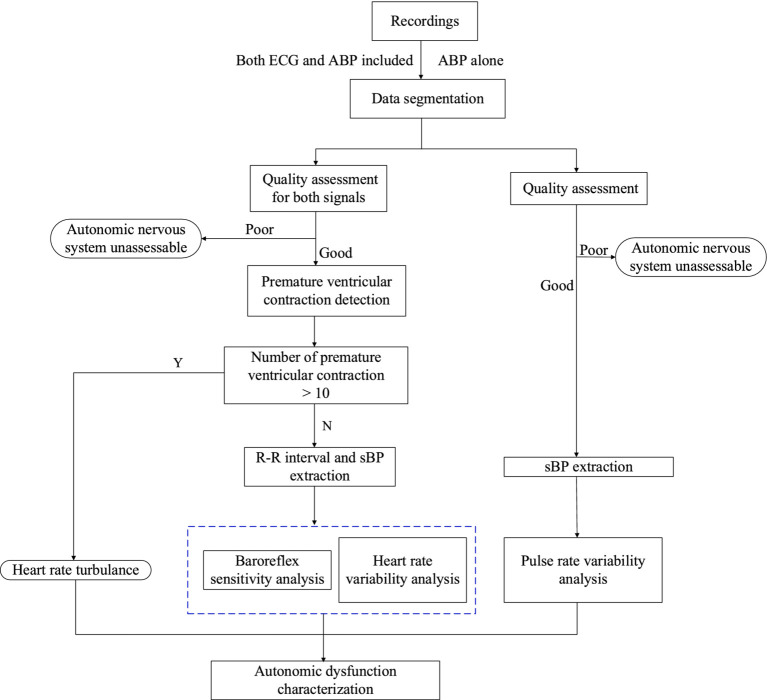
Flow diagram to compute automatic indices from 1 h ECG and ABP segments.

We will conduct non-invasive characterization of eAD by computing multiple indices over 24 h following admission, including heart rate turbulence (HRT) (18), time- and frequency-domain analyses of HRV and sequence analysis of BRS. First, we will slice the long-monitoring waveform into 1-h segments and assess the signal quality for both ECG and ABP segments. Next, the premature ventricular contractions (PVC) detection will analyze high-quality ECG segments. If more than 10 PVC beats for a given 1-h segment are detected, we will conduct HRT analysis on ECG waveforms (19). HRT is generally assessed by two parameters: turbulence onset (TO) and turbulence slope (TS). TO measures the initial acceleration and subsequent deceleration of heart rate after a PVC, while TS quantifies the rate of heart rate return to baseline. We will extract two time series from the ECG and ABP waveforms: one consisting of RR intervals from normal sinus beats, and the other comprising systolic blood pressures (sBP) from ABP pulses. We will then perform three analyses on these sequences: extracting root mean square of successive differences (RMSSD) and HRT from time-domain RR interval sequences, transforming RR intervals into the frequency domain to calculate the low- and high-frequency band ratio, and estimating BRS indices from RR intervals and sBP sequences. For eAD characterization based on ABP signals alone, we will compute autonomic indices from pulse interval sequences, excluding HRT due to the difficulty of detecting PVC beats without ECG waveforms. Figure 1 summarizes the entire procedure, with detailed explanations of each function block provided in the following sections.

#### 2.2.1 Signal quality assessment

The collected waveforms may include uncontrollable noise from sensor circuits, body motion, and poor electrode attachment, making it nearly impossible to perform a reliable beat detection of such contaminated ECG and ABP waveforms. Therefore, it is essential to assess the signal quality to ensure that the eAD is characterized promisingly on high-quality waveforms.

We will assess ECG signal quality and select the lead with the best quality, based on our previous work (20). Comparable with the visual quality assessment of multi-lead ECG signals that clinicians traditionally use, we will plot collective multi-lead ECG signals in the standard paper-ECG format with grid marks and format as a multi-lead ECG image. Then, we will construct ECG image templates of two groups (good quality and poor quality) from the training database, which will be achieved by the agglomerative hierarchy cluster analysis (21) based on the structural similarity measure (SSIM).

Suppose we have a pair of ECG image $X={\left[x_{1}^{T},\ldots ,x_{M}^{T}\right]}^{T}$ and $Y={\left[y_{1}^{T},\ldots ,y_{M}^{T}\right]}^{T}$ , where $x_{m},y_{m}\in R^{N}$ , m=1,…,M , and M is number of leads. The SSIM between two single lead ECG x and y can be calculated as Equations 1, 2


(1) $$\mathrm{SSIM}\left(x,y\right)=\frac{\left(2\mu_{x}\mu_{y}+C_{1}\right)\left(2\partial_{xy}+C_{2}\right)}{\left(\mu_{x}^{2}+\mu_{y}^{2}+C_{1}\right)\left(\partial_{x}^{2}+\partial_{y}^{2}+C_{2}\right)}$$

where


(2) $$C_{1}={\left(K_{1}L\right)}^{2},C_{2}={\left(K_{2}L\right)}^{2},K_{1}<<1,K_{2}>>1$$

$\mu_{x},\mu_{y},\partial_{x},\partial_{y}$ and ${б}_{xy}$ are means, standard deviations and cross-covariance of x and y . $C_{1}$ and $C_{2}$ are constants depending on the dynamic range of each ECG sample. For each ECG pair with M leads, we will have a total of M×M values and use the mean value as final similarity. All similarity values are divided into two groups using 2-means clustering, corresponding to good and bad quality, respectively. For an ECG image cluster containing a total of T images, the representative one with index c can be choose by Equation 3


(3) $$\begin{array}{c} c=\underset{j\in \left[1,N\right]\;}{\mathrm{argmax}}\frac{\sum_{i=1}^{N}S\left(I_{i},I_{j}\right)}{N}\;\\ \;\;\;\; \end{array}$$

where N is the number of images in the cluster, and $S\left(I_{i},I_{j}\right)$ is the SSIM result between the image $I_{i}$ and $I_{j}$ within each cluster. Lastly, we format the result of SSIM between each test ECG image and template images as the input features to the linear discriminant analysis classifier to determine ECG quality.

For multi-lead ECG recordings with good quality, we will further select the optimal ECG lead. We will calculate several signal quality indices (SQIs) for each lead from a good-quality recording as introduced in published works (22, 23): (1) the consensus beat detection signal quality index (bSQI), which measures the percentage of beats detected by two beat detection algorithms; (2) the spectral distribution SQI (sSQI), which calculates the proportion of the spectral distribution of a given ECG segment found to be within a certain physiological frequency band; (3) the kurtosis-based SQI (kSQI), which evaluates the kurtosis of a segment. Then, we will combine these individual SQIs into a composite signal quality index to select the optimal lead based on expert knowledge.

We will assess ABP signal quality using a singular value decomposition (SVD)-based approach (24). We will use our previously developed pulse detection algorithm to mark the onset of each ABP pulse, normalizing each pulse in both time and amplitude (25). Once the onsets of pulses are identified, we will adjust each pulse to a fixed length using spline resampling to ensure consistency in the number of data points across all pulses. Additionally, we will standardize each pulse’s amplitude by centering it around zero and scaling it to a standard deviation of one. Then, we will project this ABP pulse onto the signal subspace based on the SVD approach and calculate the signal-to-noise ratio. To obtain the signal and noise subspaces, we will implement SVD approach on an expert-validated reference library of 567 ABP pulses collected from 51 patients hospitalized at UCLA Ronald Reagan medical center. These valid ABP pulses will be subjected to the same normalization process. We will define the signal subspace and noise subspace after conducting SVD valid ABP pulses in the reference library. For a test ABP pulse, we will first project it onto the signal subspace and calculate the ratio of the energy of the projected signal over that of the projected noise. This ratio will be compared with a threshold, which is defined as the minimum calculated ratio among all the 567 pulses in the reference ABP library. This setting ensures that any ABP pulse closely resembling any pulse in the validated library is assessed correctly, providing a robust measure against false detections. The valid ABP pulses are those with calculated ratio greater than the threshold. We will conduct subsequent analysis only on the one-hour segments that pass both ECG and ABP signal quality assessments.

#### 2.2.2 Peaks detection

We will recognize R peaks for each ECG beat using a published QRS detection algorithm that includes four steps (26). The first step will use a sliding window of 2 s to capture the ECG signal. In the second step, we apply a band-pass filter ranging from 0.5 to 17 Hz to eliminate signal noise and motion artifacts. Then, we apply an enhancement mask to the filtered signal as Equation 4:


(4) $$S\left(n\right)=\overset{k}{\underset{j=-k}{\sum }}M\left(k+j\right)E\left(n+j\right)$$

The enhancement mask M is defined as Equation 5:


(5) $$\left\{\begin{array}{c} M\left(n\right)=-1,n\in \left[0,k-1\right]\\ M\left(n\right)=2*k,n=k\\ M\left(n\right)=-1,n\in \left[k+1,2k\right] \end{array}\right.$$

where E is the filtered ECG signal and S is the enhanced ECG signal. To eliminate variations in QRS amplitude, we will normalize the amplitude of the enhanced ECG signal to 1 using min-max normalization.

The third step is the detection of QRS fiducial points based on detected crests and troughs. Based on the normal range of the QRS complex in a typical lead-II ECG waveform, as shown in a published work, we will use a searching range of 0.3 s to detect the QRS complex. The searching process starts at the first point of the normalized ECG signal, and the amplitude threshold for detecting the QRS complex will be defined as 0.5 mV (26). The last step is the R peak recognition based on the detected QRS fiducial point. Since a normal QRS rangs is less than 0.12 s, the time interval for recognizing R peak is set as 0.24 s centering at the fiducial point in (26), and we identify the R peak successively.

For the high-quality ABP waveform, we will detect systolic blood pressure based on a published algorithm (27). Initially, we will apply a Savitzky–Golay filter to remove noise. Subsequently, we will utilize a sliding window to correctly identify the maximum point of the arterial pulse.

#### 2.2.3 PVC detection

Because HRT that occurs after a premature ventricular contraction (PVC) can reflect the condition of the autonomic nervous system (28), detection of PVC beats is for subsequent HRT calculation. To reduce the computation cost, we will primarily classify ECG signals into PVC or non-PVC segments and then detect PVC locations only on PVC segments.

We will first classify 1-h ECG signals into PVC or non-PVC segments using an existing algorithm, which employs a Siamese network architecture to capture complementary information from two-lead ECG signals (29). During training, the model will take a pair of ECG signals as inputs. In each training iteration, the two signal modalities will take turns flowing through the encoder and the projector of this network. The learned features of one ECG channel will pass through the predictor to map to the latent space of features from the other ECG channel. We will optimize an agreement loss between the predicted latent feature vector of one ECG channel ECG and the projected latent feature vector of the other ECG channel. We will also optimize a supervised cross-entropy loss for the output of the classifier function, which takes the latent features of one ECG channel as inputs. Upon completion of training, only the encoder and classifier are preserved for subsequent predictions. During testing, the trained network can operate using single lead ECG independently, without necessitating an ECG signal pair. After the PVC segments are classified, we will adopt the algorithm in (30) to identify the PVC locations in two stages. In the first stage, based on the PVC morphological characteristics in high width, large amplitude and an abnormal waveform, we identify rough PVC beats through the quantification of these qualities with rules. In the second stage, we refine detection to reject false positive and normal beats. If a solitary PVC candidate is identified after initial screening and its morphology closely aligns with that of the majority of other beats, it should be reclassified as non-PVC and excluded from further consideration. Conversely, if there are multiple PVC candidates, their widths (cW) are computed at three-fourths the height of the smallest candidate. Subsequently, the PVCs are assessed pairwise. A PVC that exhibits considerable deviation from others and possesses a cW exceeding the mean is designated as an ectopic PVC. Candidates failing to meet these criteria are categorized as non-PVC and consequently excluded from the analysis.

##### 2.2.3.1 Methods evaluation

PVC detection enables the correct identification of HRT onset and slope. To obtain the accurate location of PVCs, we will test several PVC detectors, including the Siamese network-based PVC algorithm mentioned above, using publicly available datasets and compare their performance. We will select the method that demonstrates optimal performance as the final solution for PVC detection on our collected waveform data. The evaluation process will include the following steps.

###### 2.2.3.1.1 Evaluation dataset

We will use the St Petersburg INCART 12-lead Arrhythmia database available from Physionet (31) as the evaluation dataset. This database includes multiple recordings from each of the 32 patients undergoing tests for coronary artery disease, resulting in 75 annotated recordings (17 men and 15 women, aged 18–80; mean age: 58). In selecting records for inclusion, preference was given to subjects with ECGs indicative of ischemia, coronary artery disease, conduction abnormalities, and arrhythmias. Each recording is 30 min and sampled at 257 Hz. The PVC beat is annotated by an automatic algorithm and manual correction. Because PVC is global and training model can learn from more exposures to different combinations of ECG leads, we will expand the training dataset by forming more pairs of ECG leads. Then, we will slice the ECG signals into 30 s segments with 1 s nonoverlapping. We label each 30 s segment as a PVC segment if it contains at least one PVC beat. We then apply the five-fold cross-validation splitting data based on subjects to validate the performance of the algorithm. We will use the MIT-BIH Arrhythmia database available from PhysioNet (31, 32) as the test dataset, which includes 48 2-lead ECG recordings obtained from 47 subjects (two recordings from the same subject), studied by the BIH Arrhythmia Laboratory. The subjects include 25 men aged 32 to 89 years and 22 women aged 23 to 89 years.

###### 2.2.3.1.2 Evaluation with state of the art PVC detections

Several state-of-the-art tools for PVC detection are available with open code, and we will compare them to our primary method. The first one proposes a convolutional neural network-based deep learning model, ECGDet, to detect PVC beats for every 32 points of the ECG signals (33). The second PVC detection algorithm involved two stages (34): the first classifies heartbeats into ectopic and non-ectopic beats, and the second further classifies the ectopic heartbeats into PVC beats. The last algorithm (35) combined the long short-term memory network with autoencoder to extract features of ECG heartbeats for K-means clustering and construct templates. This algorithm identifies PVC beats based on the similarity with these templates. To evaluate the performance of PVC detection, we will use sensitivity (Sen), specificity (Spec), accuracy (Acc) and F1-score. The definitions of these four metrics are provided in the Appendix.

#### 2.2.4 Heart rate turbulence quantification

HRT refers to the fluctuations of sinus heart beat cycle length caused by PVC beats (18). When a PVC beat occurs, it results in a shortened interval with the prior beats and this interval is termed coupling interval (couplI). A longer interval than that of a normal sinus beat is then followed, which is a compensatory interval (compI) (36). The HRT reflects the natural fluctuation of HR after the compI. If more than 10 beats are detected as PVC, we will conduct HRT analysis to compute turbulence onset (TO) and slope (TS) (19) for each PVC beat. TO is an indicator to reflect the vagal inhibition by quantifying the initial fast increase of heart rate (36) and calculated as Equation 6:


(6) $$TO=\frac{\left(RR_{1}+RR_{2}\right)-\left(RR_{-2}+RR_{-1}\right)}{RR_{-2}+RR_{-1}}\times 100\%$$

where $RR_{1},RR_{2}$ are two RR intervals immediately after compI, and $RR_{-1},RR_{-2}$ are two RR intervals immediately preceding couplI.

TS is an indicator to reflect the vagal activation and is measured as the maximum positive regression slope over any 5 consecutive sinus rhythm RR intervals within the first 15 RR intervals after the compI (19). Therefore, the analysis for both HRT parameters will require at least 2 sinus rhythm RR intervals before couplI and at least 15 RR intervals after compI. For 1-h ECG segments, we will compute the mean of TO and TS across all detected PVCs to obtain an average value that represents the overall heart rate turbulence for the recording period.

#### 2.2.5 BRS analysis

While heart rate changes due to acceleration and deceleration involve various physiological mechanisms beyond baroreceptor activity—including direct autonomic influences, mechanical factors, and other reflex pathways (37–39)**—**the combined analysis of baroreflex sensitivity (BRS) with directional heart rate changes offers a comprehensive view of autonomic regulation. This approach is particularly informative because the temporal relationship between blood pressure changes and subsequent heart rate responses helps distinguish baroreflex-mediated changes from other factors. Additionally, the magnitude and timing of these heart rate responses provide insights into baroreflex gain, even amid other regulatory influences. Analyzing both acceleration and deceleration also allows for the assessment of potential asymmetry in autonomic control, which is crucial in conditions like TBI where sympathetic and parasympathetic responses may be unevenly affected. By integrating these heart rate responses with traditional BRS measures, a more detailed understanding of cardiovascular regulation in sTBI patients is achieved, highlighting the complexities of impaired autonomic control.

We will use a phase-rectified signal averaging (PRSA) algorithm to compute BRS metrics. The process includes several steps regarding to the published paper (37):

1. Anchor points identification: if sBP values are higher than the preceding values, these values are defined as anchor points.
2. For the synchronous RR intervals (RRI), each RRI is matched to a sBP value. A window length of 15-beat R-R intervals around each anchor point provides an optimal balance between capturing complete baroreflex responses and maintaining signal stationarity. Therefore, we select 15 RR intervals around each anchor point as a segment.
3. Segments are aligned at the anchor points leading to a phase-rectification of the segments.
4. Computation of PRSA signal: the PRSA signal $X\left(i\right)$ is obtained by averaging the signals within the aligned segments, as shown in Equations 7, 8.

(7) $$X\left(i\right)=\frac{1}{P}\overset{P}{\underset{p=1}{\sum }}RRI_{n_{p}}+l,\;l=-L,-L+1,\ldots ,L\text{.}$$

(8) $$BRS=\frac{1}{4}\left[X\left(0\right)+X\left(1\right)-X\left(-1\right)-X\left(-2\right)\right]$$

where P is the total number of anchor points used in the averaging process. $RRI_{n_{p}}$ denotes the R-R interval values at the anchor point p . $n_{p}$ is the index of the anchor points with the total number of P . l is an index representing a range around the anchor points from −L to L , where L is the length of the segments before and after the anchor point considered for averaging. The expression $RRI_{n_{p}}+l$ indicates the RR interval value at the position offset by l from the $p^{th}$ anchor point. $X\left(0\right),X\left(1\right),X\left(-1\right)$ and $X\left(-2\right)$ are the PRSA signal values at offsets 0, 1, −1, and − 2, respectively.

In addition, we will estimate BRS in the frequency domain (27). We will identify an autoregressive with exogenous input (ARX) model with sBP time series as input and RR interval time series as output, which is indicated as Equation 9 (40):


(9) $$RR\left(k\right)=\overset{N}{\underset{i=1}{\sum }}a_{i}*RR\left(k-i\right)+\overset{N}{\underset{j=0}{\sum }}b_{j}*sBP\left(k-j\right)+e\left(k\right)$$

where $a_{i}$ and $b_{i}$ are coefficients of the ARX model and e is a random error with zero mean. We will then calculate the PSD of the transfer function within the LF frequency band (0.04–0.15 Hz) and the gain of the transfer function will be used as an estimate of BRS as Equation 10 (41):


(10) $$\alpha \left(LF\right)=\sqrt{\frac{P_{RR}\left(LF\right)}{P_{BP}\left(LF\right)}}\text{.}$$

where $P_{RR}\left(LF\right)$ and $P_{BP}\left(LF\right)$ indicate the spectral densities in LF band of RR interval and sBP time series, respectively.

#### 2.2.6 Heart/pulse rate variability analysis

The autonomic nervous system constantly regulates sBP and heart rate and the oscillations at different frequencies in these signals therefore reflect the status of the ANS regulation. We will extract metrics in both time domain and frequency domain. In the time domain, we will compute RMSSD between inter-beat intervals of sinus beats.

There are several reasons that we select RMSSD as the primary measure: (1) RMSSD is particularly sensitive to short-term, high-frequency variations in heart rate, making it an excellent index for capturing rapid vagal modulation of heart rate (42). (2) RMSSD demonstrates superior mathematical properties compared to pNN50, including better statistical stability when analyzing short segments of data (43). (3) RMSSD is less affected by respiratory rate changes compared to other time-domain measures, which is particularly important in mechanically ventilated TBI patients (44).

In the frequency domain, as highlighted in (45) and (42), the LF component (0.04–0.15 Hz) was previously known as the baroreceptor range, as it primarily reflects baroreceptor activity under resting conditions. LF power can be influenced by both the parasympathetic and sympathetic nervous systems, as well as by blood pressure regulation through baroreceptors. The sympathetic nervous system generally does not produce rhythms above 0.1 Hz, whereas the parasympathetic system can influence heart rhythms down to 0.05 Hz, which corresponds to a 20-s rhythm. Under resting conditions, the LF band is indicative of baroreflex activity rather than cardiac sympathetic innervation. During periods of slow breathing, vagal activity can induce oscillations within the LF band. The HF component (0.15–0.4 Hz) is closely tied to respiratory rates, which is why it is also referred to as the respiratory band. The rate of breathing modulates vagal activity through respiratory sinus arrhythmia, the natural increase and decrease in heart rate that occurs with inhalation and exhalation. Hence, the HF band is predominantly a measure of parasympathetic influence on the heart. Because observed RR interval samples are non-uniformly spaced in time, we will use the well-established Lomb-Scargle power spectral density (PSD) estimator to calculate PSDs of the RR interval sequence (46). From the calculated PSD of the RR interval sequence, we will derive the LF/HF ratio.

For the case of eAD characterization utilizing ABP signal alone, we will extract pulse-to-pulse interval (PPI) based on detected systolic blood pressure from high-quality ABP waveforms and calculate pulse rate variability. We identify the intervals between fiducial points in successive pulses as PPI. Based on PPI time series, we will calculate RMSSD, the ratio of LF/HF and BRS metrics in place of using RR interval time series.

### 2.3 Coherence analysis

Coherence analysis between pulse rate variability and blood pressure variability will be performed on 1-h ABP waveform segments. Peaks corresponding to systolic pressure of each cardiac cycle will be detected, and Gaussian filter will be applied to exclude frequencies above 10 Hz (47). Pulse rate variability and blood pressure variability will be calculated based on frequency domain analyses of the waveform derived after applying a Gaussian shape to the time differences between subsequent cardiac cycles. Power spectral density (or power spectrum) will be used to quantify the frequency content of a blood pressure variability or pulse rate variability. Coherence will be calculated using mscohere function in MATLAB (47).

## 3 Statistical analysis

The characterization of eAD requires consideration of age-specific analysis. We will stratify patients into age to account for known age-related differences in autonomic function; Within each age stratum, we will establish reference distributions based on published normative data for that specific age group; For each autonomic measure, we will use age-appropriate reference ranges from healthy controls as reported in large-scale studies. Normally, RMSSD values should fall between 19 and 75 ms (48), HRT should exhibit a turbulence onset near 0% and a turbulence slope above 2.5 ms/R-R interval (49), and BRS is around 4.87–34.07 across different age groups over 20 years old (50).

For the determination of eAD, we will quantify the incidence and magnitude of anomalies such as reduced RMSSD, HRT, and BRS, alongside increased low frequency (LF) power and an elevated LF/HF ratio, which are critical indicators. eAD will be characterized if three or more of these indicators exceed or fall below 2.5 standard deviations from age and sex-standardized values. Autonomic function measures will also be considered as continuous variables as well. For the primary analysis, we will use data from patients over their first 24 h following ICU admission to identify eAD, as well as examine the association of these measures with clinical outcomes. In exploratory analysis, we will describe autonomic function measures over the first 5 days following ICU admission.

## 4 Expected results

1. Quantification of individual eAD indices, defined as reduced RMSSD, reduced HRT, reduced BRS, decreased LF power, decreased LF/HF (45, 51) (less/greater than 2.5 SDs of age/sex-standardized values) measured over 24 h following admission.
2. Quantification of eAD, defined as 3 or more (out of 5) indices less/greater than 2.5 SDs of age/sex-standardized values measured over 24 h following admission.

## 5 Discussion

This study’s novelty lies not in the development of new autonomic measures, but rather in three key aspects: (1) the first application of HRT analysis in sTBI patients, providing new insights into post-ectopic beat autonomic regulation in this population; (2) the comprehensive integration of multiple autonomic measures in a large cohort of sTBI patients, allowing for robust characterization of autonomic dysfunction patterns specific to this population; and (3) the potential to establish normative values and clinical thresholds for autonomic measures in acute sTBI, which are currently lacking. This approach will provide clinicians with a more complete understanding of autonomic dysfunction in sTBI and its relationship to clinical outcomes.

Following sTBI, eAD is a complex and multifaceted issue that can significantly impact a patient’s overall health and recovery. Characterizing eAD is crucial for risk assessment, customizing pharmacological interventions, and assessing rehabilitation strategies. While small studies indicate that eAD is associated with poor outcomes following sTBI, the duration and full trajectory of eAD has been poorly characterized. This protocol outlines the data analysis procedure for quantifying acute autonomic dysfunction using cardiac telemetry waveforms. We will collect and annotate waveform data from 550 patients in a clinical trial and quantify the incidence and magnitude of eAD indices after the first day of admission. We will characterize eAD as the presence of 3 or more indices less/greater than 2.5 SDs of age/sex-standardized values measured over 24 h following admission.

In some patients, simultaneous ECG and ABP signals may be unavailable. To solve this problem, we will divide the collected data into subgroups with and without ECG information and examine eAD within both subgroups. We will report results of the planned study in one or multiple scientific papers in international peer-reviewed journals. We will note and deviations from this protocol, if any occur, in these publications.

When interpreting autonomic indices, particularly LF/HF ratio, several important caveats must be considered. The traditional interpretation of LF/HF as a simple marker of sympathovagal balance is problematic in acute TBI patients for several reasons. First, respiratory rate, which significantly influences HF power, is often mechanically controlled in these patients. Second, medications commonly used in TBI management, such as beta-blockers and vasopressors, directly affect autonomic modulation. Third, altered intracranial pressure and cerebral autoregulation can independently influence cardiovascular variability. Therefore, we recommend interpreting LF/HF alongside other autonomic measures and within the context of each patient’s clinical status, medication profile, and ventilatory parameters. This multi-parameter approach provides a more reliable assessment of autonomic dysfunction than relying on any single metric alone.

There are still some limitations in this study. Currently, this study incorporates both linear (time and frequency domain) and non-linear (PRSA) approaches to analyze autonomic function. The inclusion of PRSA as a non-linear method is particularly valuable as it can capture complex patterns of cardiovascular regulation that may be missed by traditional linear analyses. Future studies might benefit from incorporating additional non-linear methods such as entropy measures and detrended fluctuation analysis to provide an even more comprehensive assessment of autonomic dysfunction in severe TBI. Another limitation of our study is the inability to fully control for numerous confounding factors that can influence autonomic function in the critical care setting. While we account for age through stratification and statistical adjustment, several other important confounders remain uncontrolled. These include pharmacological interventions (such as vasopressors, analgesics, sedatives, beta-blockers, and anti-edema medications like mannitol), mechanical ventilation parameters, arterial CO2 levels, and timing of various therapeutic interventions. Future studies should consider implementing more complex statistical methods to better account for these factors.

## Appendix

RMSSD is calculated between inter-beat intervals of sinus beats using the following formula:

(1)
$$\mathrm{RMSSD}=\sqrt{\frac{1}{N-1}\left(\overset{N-1}{\underset{i=1}{\sum }}{\left(RR_{i+1}-RR_{i}\right)}^{2}\right)}$$

Sensitivity measures the proportion of true PVCs correctly identified by the algorithm, calculated as follows:

(2)
$$Sen=\frac{TP}{TP+FN}$$

where TP stands for true positives and FN indicates false negatives.

Specificity quantifies the proportion of non-PVCs correctly identified, computed as follows:

(3)
$$\mathrm{Spec}=\frac{TN}{TN+FP}$$

where TN stands for true negative; FP stands for false positives.

Accuracy represents the overall correctness of PVC detection, determined as following formula:

(4)
$$Acc=\frac{TP+TN}{N}$$

where N is the total number of instances.

F1-score provides a balanced measure of the algorithm’s precision and recall, computed as follows:

(5)
$$F1=\frac{2*\left(\mathrm{precision}*\mathrm{recall}\right)}{\left(\mathrm{precision}+\mathrm{recall}\right)}$$

where precision is the ratio of TP to the sum of TP and FP, and recall is the same as sensitivity.
